# Nuclear Hormone Receptor NHR-49 Controls Fat Consumption and Fatty Acid Composition in C. elegans

**DOI:** 10.1371/journal.pbio.0030053

**Published:** 2005-02-08

**Authors:** Marc R. Van Gilst, Haralambos Hadjivassiliou, Amber Jolly, Keith R Yamamoto

**Affiliations:** **1**Department of Cellular and Molecular Pharmacology, University of CaliforniaSan Francisco, CaliforniaUnited States of America; University of CambridgeUnited Kingdom

## Abstract

Mammalian nuclear hormone receptors (NHRs), such as liver X receptor, farnesoid X receptor, and peroxisome proliferator-activated receptors (PPARs), precisely control energy metabolism. Consequently, these receptors are important targets for the treatment of metabolic diseases, including diabetes and obesity. A thorough understanding of NHR fat regulatory networks has been limited, however, by a lack of genetically tractable experimental systems. Here we show that deletion of the Caenorhabditis elegans NHR gene *nhr-49* yielded worms with elevated fat content and shortened life span. Employing a quantitative RT-PCR screen, we found that *nhr-49* influenced the expression of 13 genes involved in energy metabolism. Indeed, *nhr-49* served as a key regulator of fat usage, modulating pathways that control the consumption of fat and maintain a normal balance of fatty acid saturation. We found that the two phenotypes of the *nhr-49* knockout were linked to distinct pathways and were separable: The high-fat phenotype was due to reduced expression of enzymes in fatty acid β-oxidation, and the shortened adult life span resulted from impaired expression of a stearoyl-CoA desaturase. Despite its sequence relationship with the mammalian hepatocyte nuclear factor 4 receptor, the biological activities of *nhr-49* were most similar to those of the mammalian PPARs, implying an evolutionarily conserved role for NHRs in modulating fat consumption and composition. Our findings in C. elegans provide novel insights into how NHR regulatory networks are coordinated to govern fat metabolism.

## Abstract 2

Deletion of the *Caenorhabditis elegans* gene *nhr- 49* causes worms to accumulate fat and die younger; but these two phenotypes are a result of distinct and separable pathways

## Introduction

Due to their ability to interact with fatty acids and other lipids, nuclear hormone receptors (NHRs), including peroxisome proliferator-activated receptors (PPARs), liver X receptor, and farnesoid X receptor, are important regulators of mammalian fat metabolism; accordingly, these small-molecule-gated transcription factors have been favored targets for therapies designed to combat diabetes, obesity, and atherosclerosis [1,2,3]. Although the molecular mechanisms employed by NHRs have been intensively probed, their pleiotropic effects on overall animal physiology are only partially understood. Thus, developing a broader range of experimental systems would be useful for characterizing the fat regulatory networks integrated by NHRs and for revealing the breadth of their physiological influence.

The study of invertebrates, such as Caenorhabditis elegans, has facilitated the elucidation and interpretation of biological networks, particularly in the context of the whole animal. However, despite the fact that C. elegans contains 284 NHR genes, compared to only 48 in mammals, there is a notable absence of worm NHRs orthologous to mammalian receptors involved in fat metabolism [4,5]. In fact, it has been suggested that fat-regulating NHRs, along with several other NHR types, were lost from the C. elegans and Drosophila lineages as these organisms simplified their body plans [6]. Conceivably, then, the biological activities associated with these NHRs may also have vanished. Therefore, it is still not clear if worm receptors mediate control over fat metabolism comparable to that of mammalian NHRs.

Whether or not C. elegans employs NHRs to regulate fat metabolism, the evolution of the C. elegans NHR family is intrinsically interesting. Only 15 of the 284 worm NHRs are members of the broadly conserved subfamilies found in mammals and other metazoans [4,5]. The biological activities of most of these “conserved” NHRs have been broadly defined, revealing roles in development, molting, dauer formation, and sex determination [7,8]. The remaining 269 “divergent” NHRs have thus far been found only in nematodes and are predicted to have originated from repeated duplication of an ancestral gene that also gave rise to the hepatocyte nuclear factor 4 (HNF4) family of receptors [6]. Although mammalian HNF4 receptors have been implicated in liver development and glucose homeostasis [9], virtually nothing is known about the functions of these 269 C. elegans “nematode-specific” HNF4-like receptors. It will be interesting to determine if these divergent NHRs have adopted novel regulatory functions or whether they carry out physiological tasks similar to those of other metazoan NHRs.

Elucidating the physiological responsibilities of even one of the worm HNF4-like receptors will likely advance our understanding of NHR-regulated gene networks and provide insight into the evolution and diversification of the NHR family in nematodes. To this end, we have been systematically investigating the function of HNF4-like receptors in *C. elegans.* In this study, we present a physiological characterization of *nhr-49* (K10C3.6), one of the C. elegans receptor genes most closely related to the mammalian HNF4. Our findings reveal a surprising role for this HNF4-like receptor in the regulation of fat storage and metabolism.

## Results

### *nhr-49* Is Necessary for Normal Life Span

**Figure 1:**
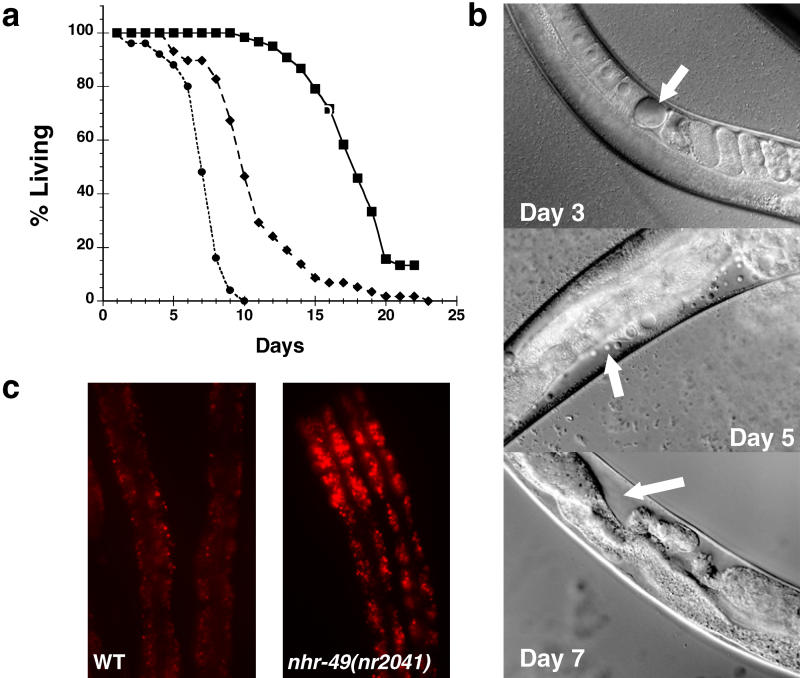
*nhr-49(nr2041)* Animals Have Reduced Life Span and Higher Fat Content (A) Adult life span of WT (black squares, solid line), *nhr-49(nr2041)* (black circles, dotted line), and *nhr-49* RNAi animals (black diamonds, dashed line). (B) Nomarksi images of *nhr-49(nr2041)* at days 3, 5, and 7 of adulthood. The arrow in the day 3 image points to a gonadal vacuole that is typical of day 3 worms; the arrow in the day 5 image shows the continued deterioration of oocytes in the gonad, and the arrow in the day 7 image points to the clearing that results from complete gonadal necrosis and collapse. (C) Nile Red intestinal fat staining of WT and *nhr-49(nr2041).* Each image displays two representative worms from a population of L4 animals.

In an RNA interference (RNAi) screen designed to identify the role of C. elegans HNF4-like receptors in worm development and longevity, we found that interference of *nhr-49* resulted in dramatically reduced life span (Figure 1A). For further characterization, we obtained a C. elegans strain, *nhr-49(nr2041),* which harbors a deletion in the *nhr-49* gene encompassing part of the DNA binding domain and more than half of the ligand binding domain (LBD); this deletion likely results in complete loss of function [10]. At 23 °C, *nhr-49(nr2041)* worms lived only 6–8 d as adults, significantly shorter than the 15 to 18-d life span of N2 wild-type (WT) animals (Figure 1A). Although *nhr-49* deletion did not noticeably affect development or fertility, *nhr-49(nr2041)* worms experienced rapid decline in function beginning around day 3 of adulthood, when vacuoles appeared in the intestine and gonad (Figure 1B). By days 4 and 5, *nhr-49(nr2041)* animals were significantly smaller than WT, vacuoles were ubiquitous, and there was widespread gonadal necrosis. By days 5–7, the gonad had completely deteriorated, and the worms died shortly thereafter. Thus, *nhr-49* function is not required for development or fertility, but is clearly essential for normal longevity. Even though the increased vacuole formation was consistent with reported aging characteristics [11], we have not determined whether the shortened life span of *nhr-49(nr2041)* reflects accelerated aging or an unrelated pathology.

### *nhr-49(nr2041)* Animals Display Abnormally High Fat Content

*nhr-49* was recently identified in a C. elegans genomewide RNAi screen as one of 112 genes that, when knocked down by RNAi, resulted in abnormally high Nile Red fat staining [12]. To determine whether deletion of *nhr-49* also yielded a high-fat phenotype, we used a similar Nile Red assay to visualize fat content in *nhr-49(nr2041).* Indeed, we found that *nhr-49(nr2041)* animals stained more brightly with Nile Red than did WT worms (Figure 1C). The difference in fat content between WT and *nhr-49(nr2041)* animals was most pronounced in the L3 and L4 stages of larval development; by day 2 of adulthood there was only a slight change in Nile Red staining, suggesting that the effects of *nhr-49* on visible fat content may vary during development (data not shown).

### *nhr-49* Regulates Genes Involved in Energy Metabolism

**Figure 2:**
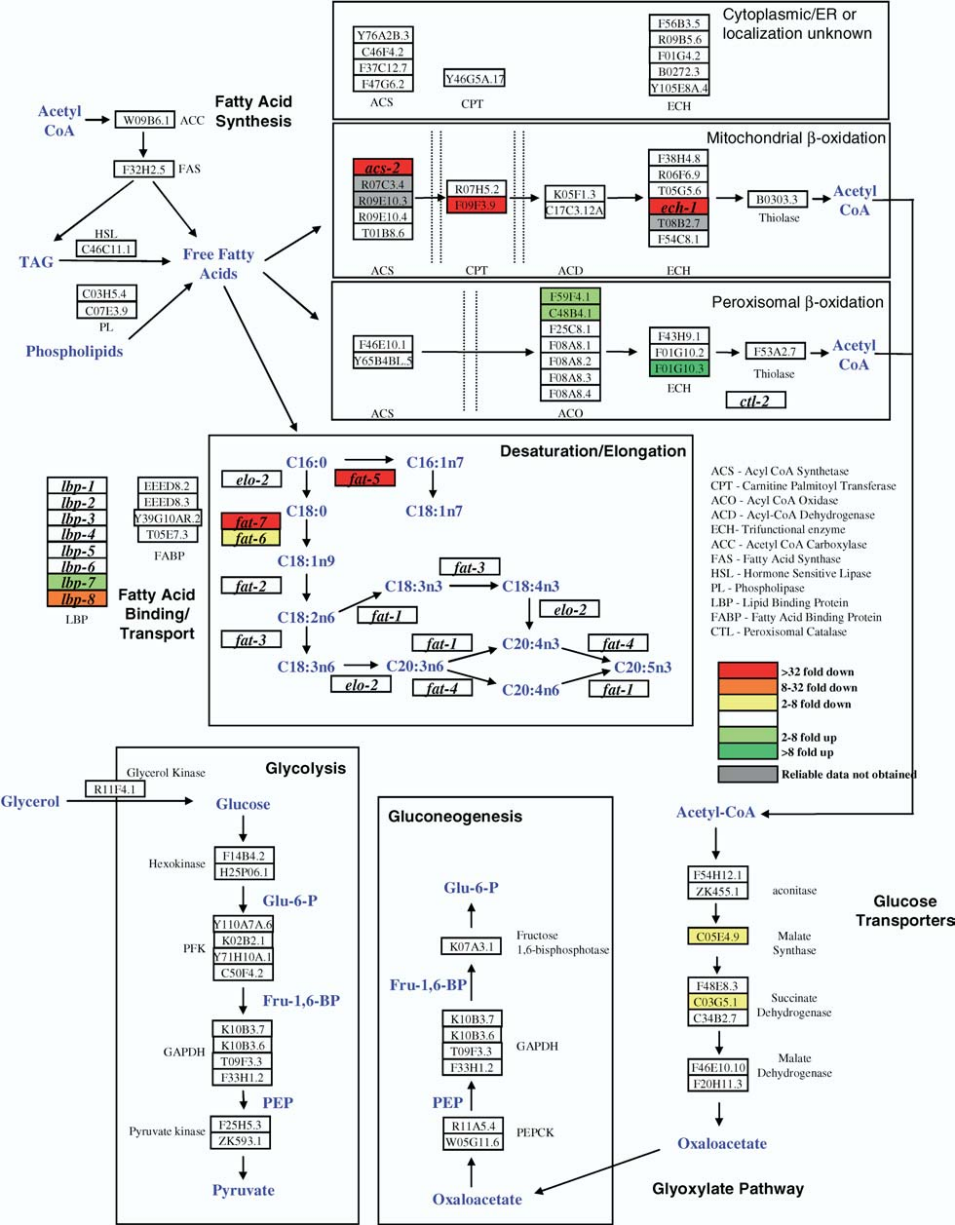
*nhr-49* Deletion Affects the Expression of 13 Energy Metabolism Genes Putative C. elegans fatty acid, glucose, and glyoxylate metabolism pathways are shown here; each box represents a gene predicted by sequence homology to code for the enzyme indicated in the figure. The fatty acid desaturation elongation pathway is adapted from previously published studies [14,17]. Mitochondrial localization of β-oxidation enzymes was predicted by TargetP and PSORT (see Materials and Methods); peroxisomal β-oxidation enzymes all contain a carboxy-terminal peroxisomal localization signal. QRT-PCR was used to measure the expression of these 89 genes in WT and *nhr-49(nr2041).* Genes expressed at lower levels in *nhr-49(nr2041)* are shown in red (>32-fold lower); orange (8- to 32-fold lower); or yellow (2- to 8-fold lower); and genes expressed at higher levels are shown in light green (2- to 8-fold higher) or dark green (>8-fold higher). Online QRT-PCR data for this analysis are available in Table S1.

The high-fat phenotype of the *nhr-49(nr2041)* mutant led us to suspect that *nhr-49* might regulate genes involved in energy metabolism. To test this hypothesis, we identified from a survey of the C. elegans genome 65 genes predicted to participate in fatty acid synthesis, β-oxidation, desaturation, elongation, and binding/transport, in addition to 16 genes expected to function in glycolysis, gluconeogenesis, and glucose transport, and eight genes involved in the glyoxylate pathway (Figure 2). We then employed quantitative RT-PCR (QRT-PCR) to measure the expression of all 89 of these genes in WT and *nhr-49(nr2041)* mutant worms. For the purpose of our survey, gene expression was measured in all four larval stages (for a complete dataset, see Table S1).

Our screen revealed that deletion of *nhr-49* significantly altered the expression of 13 genes, including six genes predicted to be involved in fatty acid β-oxidation, three genes involved in fatty acid desaturation, two genes involved in fatty acid binding/transport, and two genes involved in the glyoxylate pathway (Figure 2). Similar changes in gene expression were observed when *nhr-49* was knocked down in WT animals using RNAi (data not shown).

Overall, *nhr-49* deletion displayed the most dramatic effects on two metabolic pathways, mitochondrial β-oxidation and fatty acid desaturation, as the expression of multiple genes in each of these pathways was significantly compromised by loss of *nhr-49* function (Figure 2). In contrast, *nhr-49* deletion had smaller and mixed consequences for genes that participate in peroxisomal β-oxidation and lipid binding/transport. *nhr-49* knockout also marginally reduced expression of two enzymes involved in the glyoxylate pathway, a pathway that facilitates the conversion of fatty acid β-oxidation products to glucose (Figure 2). *nhr-49* deletion did not, however, significantly affect the expression of any genes predicted to be involved in glucose metabolism. Thus, it is clear that *nhr-49* is extensively involved in the control of fatty acid metabolism, with a pronounced role in the promotion of mitochondrial β-oxidation and fatty acid desaturation.

### *nhr-49(nr2041)* Animals are High in Fat Due to Reduced Expression of β-Oxidation Genes

**Figure 3:**
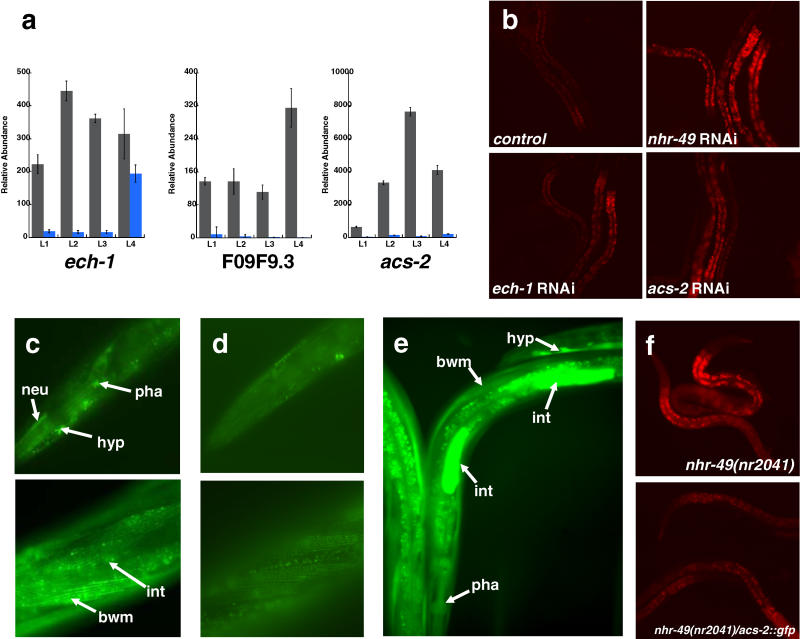
Regulation of β-Oxidation Gene Expression by *nhr-49* (A) QRT-PCR measurement of *acs-2,* F09F9.3, and *ech-1* expression in WT (gray bars) and *nhr-49(nr2041)* animals (blue bars). Expression was measured in all four stages of larval development. Error bars represent standard error of measurement. (B) RNAi of *nhr-49, acs-2,* or *ech-1* resulted in increased Nile Red fat staining. Each image shows 3 or 4 representative worms from a population of L4 animals grown on plates containing RNAi bacteria and Nile Red fat-staining dye. (C) *acs-2::gfp* is expressed in many tissues including hypodermis (hyp), intestine (int), body wall muscle (bwm), neurons (neu), and pharynx (pha). ACS-2::GFP localizes to subcellular structures in a pattern similar to what has been reported for mitochondrial proteins [13]. (D) Expression of *acs-2::gfp* was lower in *nhr-49(nr2041)* mutant animals. (E) Expression of P*_nhr-49_::gfp* promoter fusion in WT animals revealed that *nhr-49* is expressed in multiple tissues, including hypodermis (hyp), body wall muscle (bwm), pharynx (pha), and intestine (int). The animals shown here are genetically mosaic, harboring the P*_nhr-49_::gfp* construct in only a fraction of total cells. (F) Ectopic expression of *acs-2::gfp* in *nhr-49(nr2041)* was sufficient to reduce Nile Red staining to WT levels.

Three genes predicted to participate in mitochondrial β-oxidation, *ech-1* (C29F3.1), F09F3.9, and an acyl-CoA synthetase gene *(acs-2)* (F28F8.2), were expressed at significantly lower levels in *nhr-49(nr2041)* animals throughout development (Figure 3A). *acs-2* is predicted to encode a mitochondrial acyl-CoA synthetase, whereas F09F3.9 likely codes for a carnitine palmitoyl transferase, and *ech-1* appears to encode a mitochondrial β-oxidation trifunctional enzyme. Because mitochondrial β-oxidation facilitates the degradation of stored fats for the production of energy, we suspected that *nhr-49(nr2041)* worms might be high in fat due to the reduced expression of these key β-oxidation enzymes. To test this hypothesis, we used RNAi to reduce separately the expression of *acs-2,* F09F3.9, and *ech-1* in WT animals. Indeed, we found that RNAi of either *acs-2* or *ech-1* resulted in worms with elevated Nile Red fat staining (Figure 3B). *acs-2* was previously identified for a similar effect on fat storage [12]. We conclude that *nhr-49* promotes the expression of three β-oxidation genes, two of which, in WT worms, reduce overall fat storage. Thus, *nhr-49(nr2041)* mutant worms are likely to be high in fat due to the impaired expression of these genes.

### ACS-2 Is Expressed in Multiple Tissues and Localizes to Mitochondria

Out of five predicted mitochondrial *acs* genes in *C. elegans, nhr-49* affected only the expression of *acs-2,* suggesting that *nhr-49* may only influence a subset of mitochondrial β-oxidation pathways in worms. To determine if *acs-2* was expressed in a specific set of tissues, we fused the full-length *acs-2* gene and promoter to *gfp (acs-2::gfp);* injection of this construct into WT worms revealed that the ACS-2::green fluorescent protein (GFP) was widely expressed in many cell types, including intestine, hypodermis, pharynx, body wall muscle, and several neurons (Figure 3C). Moreover, ACS-2::GFP expression was reduced in all of these tissues in *nhr-49(nr2041)* worms, indicating that the effects of *nhr-49* on *acs-2* expression were widespread (Figure 3D). Notably, the expression pattern of *acs-2* overlapped extensively with that of an *nhr-49* promoter/GFP fusion, consistent with the idea that *nhr-49* could control *acs-2* through direct transcriptional activation (Figure 3E). As predicted, ACS-2::GFP localized to subcellular structures in patterns similar to those reported for other mitochondrial proteins [13] (Figure 3C).

### Overexpression of ACS-2::GFP Is Sufficient to Suppress the High-Fat Phenotype of *nhr-49(nr2041)*

Strikingly, overexpression of the ACS-2::GFP fusion was able to suppress the high-fat phenotype of *nhr-49(nr2041)* animals (Figure 3F). These data suggest that *acs-2* expression is reduced in *nhr-49(nr2041)* such that it becomes a rate-limiting factor in the consumption of fat; thus, high fat levels can be overcome simply by increasing *acs-2* expression. Overexpression of *acs-2* in WT animals did not affect fat content, however, indicating that WT levels of *acs-2* expression are not rate limiting. These data further support the conclusion that reduced expression of *acs-2* is a significant contributor to the high-fat phenotype of the *nhr-49(nr2041)* mutant.

### *nhr-49* Modulates Fatty Acid Composition by Promoting Expression of Δ9-Desaturases

**Figure 4:**
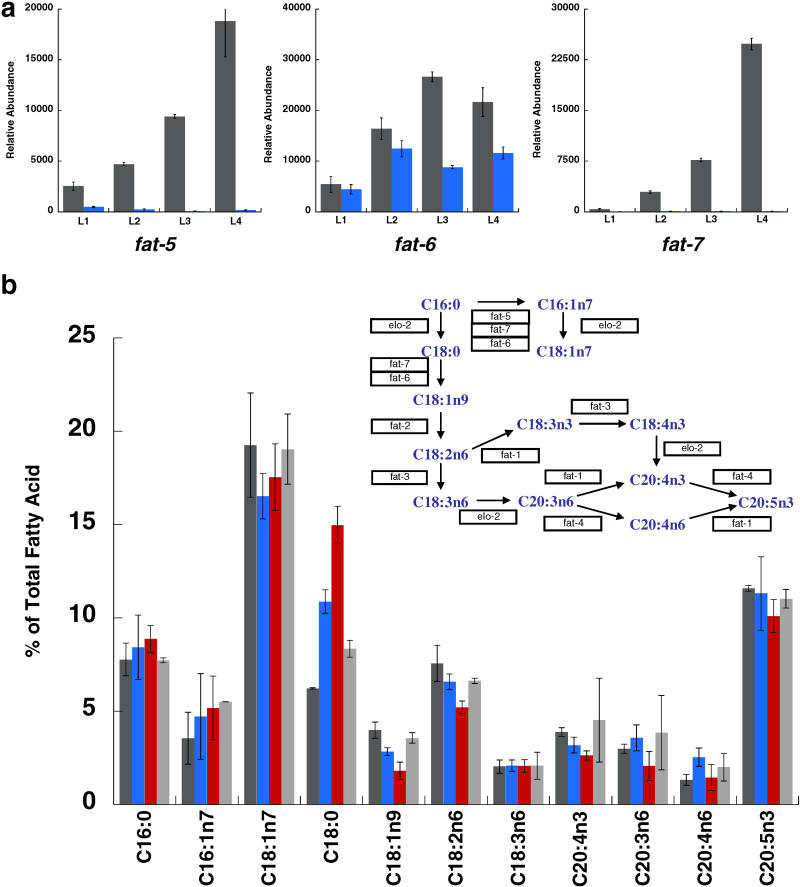
Deletion of *nhr-49* Hinders Fatty Acid Desaturation (A) QRT-PCR measurement of *fat-5, fat-6, and fat-7* expression in WT (gray bars) and in *nhr-49(nr2041)* mutant worms (blue bars). Expression was measured in all four stages of larval development. Error bars represent standard error of measurement. (B) Relative abundance of individual fatty acid species expressed as percentage of total measured fatty acid. Fatty acids included in total fatty acid measurement but not shown in the figure include C14:0, C15:0, and C17:Δ. Fatty acids were isolated and quantified by GC/MS from WT (dark gray bars), *nhr-49(nr2041)* (blue bars), *fat-7* RNAi animals (red bars), and *nhr-49* RNAi animals (light gray bars). Error bars represent standard error. The fatty acid desaturation/elongation pathway, along with enzymes involved in desaturation and elongation, is shown in the inset; this pathway was adapted from previous studies [14,17].

In addition to stimulating genes in the mitochondrial β-oxidation pathway, *nhr-49* also promoted the expression of three C. elegans fatty acid desaturases (see Figure 2). *fat-5* (W06D12.3) and *fat-7* (F10D2.9) expression was dramatically lowered in *nhr-49(nr2041)* worms (>30-fold) in all four larval stages, whereas *fat-6* (VZK8221.1) expression was marginally reduced (approximately 2-fold) only in L3 and L4 animals (Figure 4A).

Studies in yeast have shown that the *C. elegans fat-5, fat-6,* and *fat-7* genes encode Δ9-desaturases, which preferentially convert saturated C16:0 and C18:0 fatty acids to monounsaturated C16:1 and C18:1 fatty acids [14]. *fat-5* is a palmitoyl-CoA desaturase, specifically acting on palmitic acid (C16:0), and *fat-6* and *fat-7* are stearoyl-CoA desaturases (SCDs), preferentially functioning to desaturate stearic acid (C18:0). Mammalian SCDs have been shown to be important for maintaining an appropriate level of fatty acid desaturation, vital for modulating membrane fluidity and for controlling lipid metabolism [15,16]. Furthermore, in *C. elegans,* SCDs are also involved in catalyzing the first step in the synthesis of polyunsaturated fatty acids (PUFAs) [17] (see Figure 4B inset).

Because the expression of these three C. elegans Δ9-desaturases was significantly impaired by *nhr-49* deletion, we used gas chromatography/mass spectrometry (GC/MS) to determine whether overall fatty acid desaturation and PUFA synthesis were altered in *nhr-49(nr2041)* animals. The most significant consequence of *nhr-49* deletion on fatty acid composition was a marked increase in the abundance of stearic acid (C18:0) and a corresponding decrease in the level of oleic acid (C18:1n9) (Figure 4B). This result is consistent with a reduction in SCD activity.

In mammals, a primary role of SCD is to control the ratio of fully saturated stearic acid to monounsaturated oleic acid (C18:0/C18:1n9), and this ratio has been employed as an indicator of the activity of the mammalian SCD1 [15,16]. In C. elegans we found that the ratio of stearic acid to oleic acid (C18:0/C18:1n9) was increased from 1.9 in WT animals to 4.3 in *nhr-49(nr2041)* (Table 1). A similar but less pronounced alteration in the ratio of stearic acid to oleic acid was observed when *nhr-49* was knocked down in WT animals using RNAi (Figure 4B and Table 1). This smaller influence on fatty acid desaturation likely resulted from the fact that *nhr-49* RNAi did not reduce Δ9-desaturase expression as dramatically as *nhr-49* knockout (data not shown).

**Table 1:**
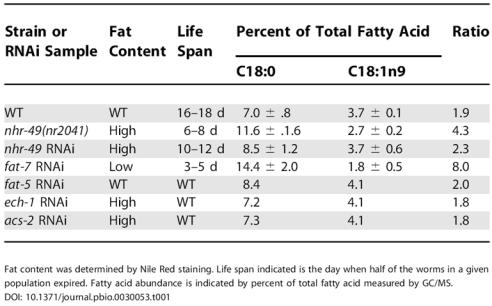
Life Span, Fat Content, and Relative C18:0 Fatty Acid Abundance

Fat content was determined by Nile Red staining. Life span indicated is the day when half of the worms in a given population expired. Fatty acid abundance is indicated by percent of total fatty acid measured by GC/MS

C. elegans SCDs are also important for the synthesis of PUFAs; however, we did not observe any measurable changes in PUFA abundance in *nhr-49(nr2041)* animals (Figure 4B). Therefore, despite the dramatic effect of *nhr-49* on SCD activity, *nhr-49* does not appear to play an appreciable role in overall PUFA synthesis.

To determine which of the Δ9-desaturases accounted for the influence of *nhr-49* deletion on fatty acid composition, we used RNAi to individually knock down expression of *fat-5* and *fat-7* (because *fat-6* expression is only reduced by approximately 2-fold in *nhr-49[nr2041],* we did not examine *fat-6* RNAi animals). Although we did not observe a significant consequence of *fat-5* RNAi on fatty acid desaturation, *fat-7* RNAi altered fatty acid composition in a manner very similar to that of *nhr-49* deletion (Figure 4B and Table 1). *fat-7* RNAi animals displayed a significant increase in the abundance of C18:0 fatty acid, as well as a decrease in the levels of C18:1n9 and C18:2n6 fatty acids. The influence of *fat-7* RNAi on fatty acid composition was stronger than that of *nhr-49* deletion, increasing the ratio of stearic to oleic acid (C18:0/C18:1n9) to 8.0. *fat-7* RNAi animals also exhibited a slight lowering of PUFA abundance. The stronger effect of *fat-7* RNAi on fatty acid composition suggests that *fat-7* expression was reduced to levels even lower than those observed in *nhr-49(nr2041).* Because *fat-7* shares significant homology (approximately 85% nucleotide identity) with *fat-6,* it was possible that *fat-7* RNAi also partially interfered with *fat-6* expression; however, we found by QRT-PCR that the overall levels of *fat-6* mRNA were not noticeably reduced by *fat-7* RNAi. (data not shown).

Therefore, we conclude that by strongly promoting SCD expression, *nhr-49* influences fatty acid composition in *C. elegans.* Although it appears that the primary effect of *nhr-49* deletion is a modulation of stearic and oleic acid levels, not PUFA abundance, we cannot rule out the possibility that *nhr-49* influences PUFA synthesis in a more localized manner, such that the changes in PUFA composition are not detectable by our whole-worm analysis.

### Interference of *fat-7* Expression Reproduces Shortened Life-Span Phenotype

**Figure 5:**
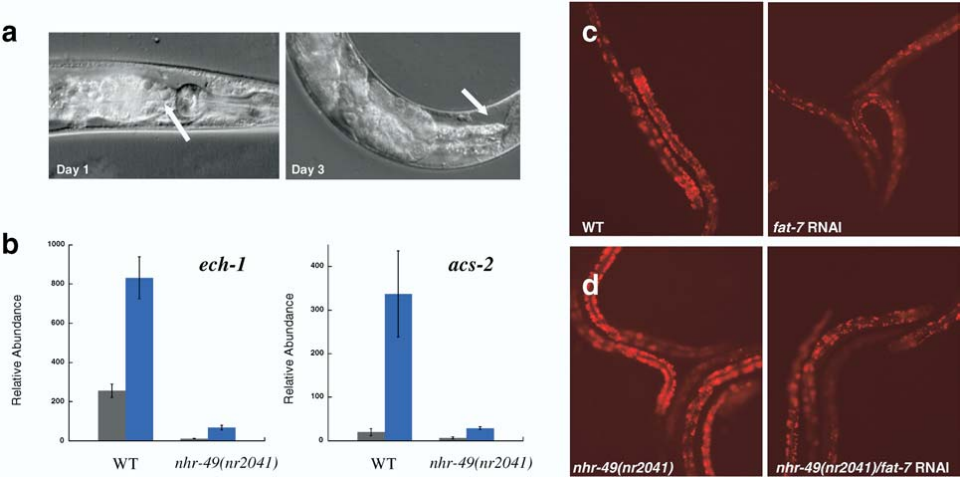
*fat-7* Is Necessary for Normal Life Span and Inhibits β-Oxidation (A) Nomarski images of WT worms subjected to *fat-7* RNAi at days 1 and 3 of adulthood. The arrow in the day 1 image points to vacuole formation in the intestine, and the arrow in the day 3 image points to clearing that results from collapse of the gonad. These characteristics are nearly identical to those observed for *nhr-49(nr2041)* worms (see Figure 1B). (B) QRT-PCR measurement of *acs-2* and *ech-1* expression in WT and *nhr-49(nr2041)* L4 animals grown on control RNAi bacteria (dark gray bars) or on *fat-7* RNAi bacteria (blue bars). Error bars represent standard error of measurement. (C) RNAi knockdown of *fat-7* expression in WT animals reduced Nile Red fat staining (D) RNAi of *fat-7* in *nhr-49(nr2041)* also decreased fat staining.

Using RNAi to knock down the *nhr-49*-dependent genes identified in this study, we found that interference of only *fat-7* resulted in a shortened life-span phenotype similar to that of *nhr-49(nr2041)* (see Table 1). *fat-7* RNAi animals displayed many of the same characteristics of the *nhr-49(nr2041)* mutant, including widespread vacuole formation and germ line necrosis (Figure 5).

Interestingly, the effect of *fat-7* RNAi on life span was even more potent than *nhr-49* deletion, reducing adult life span to 3–5 d, as opposed to the 5- to 7-d life span of *nhr-49(nr2041)* (see Table 1). This is consistent with the finding that SCD activity is more dramatically compromised in *fat-7* RNAi animals than it is in the *nhr-49(nr2041)* mutant. In fact, we observed a striking correlation between *fat-7* activity, as determined by the ratio of stearic to oleic acid in our various mutants, and life span (see Table 1). WT animals (stearic/oleic = 1.9) lived approximately 16–18 d, *nhr-49 RNAi* animals (stearic/oleic = 2.3) lived approximately 10–12 d, *nhr-49(nr2041)* animals (stearic/oleic = 4.3) lived approximately 6–8 d, and *fat-7* RNAi animals (stearic/oleic = 8.0) lived approximately 3–5 d.

Because *fat-7* expression is significantly compromised by *nhr-49* deletion, and because lowered *fat-7* expression can lead to shortened life span, we suggest that the reduced life-span phenotype of *nhr-49(nr2041)* likely reflects, at least in part, diminished *fat-7* expression.

### Stimulation of *fat-7* by *nhr-49* Feeds Back to Inhibit β-Oxidation

By promoting β-oxidation gene expression, *nhr-49* stimulates fat consumption, and by enhancing *fat-7* expression, *nhr-49* modulates fat desaturation and ensures normal longevity. We next set out to determine whether *nhr-49* regulates these two pathways independently.

Although RNAi of *acs-2* or *ech-1* was sufficient to cause a high-fat phenotype, *acs-2* or *ech-1* interference did not significantly impact life span, fatty acid composition, or desaturase gene expression, demonstrating that the effects of *nhr-49* deletion on *fat-7* expression and life span were not a downstream consequence of compromised β-oxidation or increased fat content (see Table 1).

**Figure 6:**
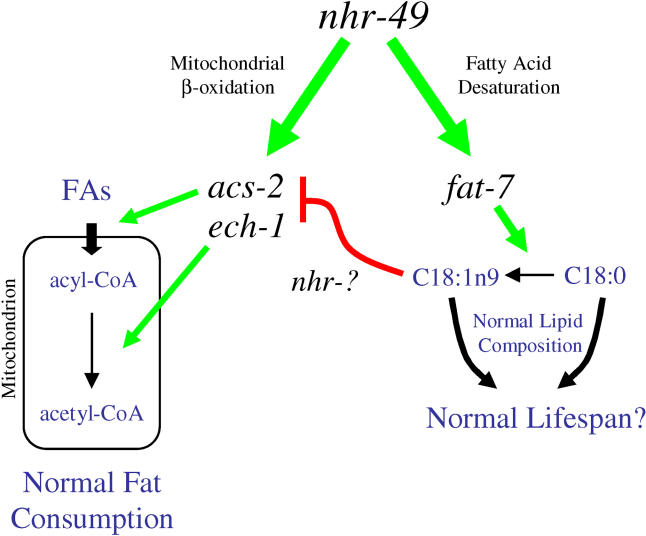
Model for *nhr-49* Regulation of Fat Storage and Composition By stimulating expression of genes predicted to be involved in fatty acid β-oxidation, *nhr-49* promotes fat consumption; likely by facilitating the flow of fatty acids (FA) into the mitochondrial matrix. Through an independent pathway, *nhr-49* modulates fatty acid desaturation by enhancing the expression of the *fat-7* SCD. Stimulation of *fat-7* is necessary for normal adult life span. Finally, *fat-7* also feeds back to partially inhibit expression of β-oxidation genes.

In contrast, RNAi of *fat-7* significantly impacted fat storage and β-oxidation. The effect was paradoxical, however, as *fat-7* interference produced the opposite result of *nhr-49* deletion, causing *reduced* fat content and *increased* expression of genes in β-oxidation, including *acs-2* and *ech-1* (Figure 5). As the influence of *fat-7* on the expression of β-oxidation genes is opposite that of *nhr-49,* it is clear that the stimulation of β-oxidation by *nhr-49* does not result from promotion of *fat-7* expression. In fact, by enhancing *fat-7* expression, *nhr-49* indirectly causes inhibition of the very same β-oxidation genes that it is independently stimulating (Figure 6).

Because *fat-7* targets some of the same β-oxidation genes as *nhr-49,* we wondered if the effects of *fat-7* on fat storage might be dependent on *nhr-49;* for example, perhaps *fat-7* represses *ech-1* and *acs-2* expression by blocking the *nhr-49* mediated induction of these genes. However, we found that both *ech-1* and *acs-2* were induced when *fat-7* was knocked down even further in the *nhr-49(nr2041)* mutant using RNAi, indicating that *fat-7* hinders the expression of *ech-1* and *acs-2* through a mechanism that is independent of *nhr-49* (see Figure 5B). Furthermore, RNAi of *fat-7* reduced fat content in *nhr-49(nr2041)* worms, demonstrating that the stimulation of fat storage by *fat-7* is also not dependent upon *nhr-49* (see Figure 5D).

Taken together, our findings demonstrate that *nhr-49* controls two distinct circuits within a gene network: The stimulation of β-oxidation gene expression is independent of *fat-7,* and the promotion of *fat-7* expression is independent of *nhr-49*'s effects on β-oxidation. However, because *fat-7* expression inhibits the same β-oxidation genes induced by *nhr-49,* the regulation of *fat-7* by *nhr-49* connects these two circuits into a single regulatory network, serving to limit β-oxidation through an apparent feedback mechanism (Figure 6).

## Discussion

Here we demonstrated that deletion of the *C. elegans nhr-49* gene produced two global phenotypes: shortened life span and high fat content. Using a QRT-PCR strategy, we found 13 genes involved in energy metabolism that depend upon *nhr-49* for expression. The phenotypes of *nhr-49(nr2041)* were attributable to deficiencies in two different metabolic pathways, fatty acid β-oxidation and fatty acid desaturation. The high-fat phenotype is explained by lowered expression of β-oxidation enzymes, and the shortened life-span phenotype results from reduced expression of the *fat-7* SCD. Interestingly, these pathways are linked by an apparent feedback mechanism, as *fat-7* also serves to inhibit the expression of fatty acid β-oxidation genes (Figure 6).

These results represent the first detailed characterization of a divergent HNF4-like receptor in C. elegans and provide the first description of an NHR-controlled fat-regulatory network in invertebrates. Due to their evolutionary relationship to *nhr-49,* it seems conceivable that many of the C. elegans HNF4-like NHRs might participate in the regulation of fat metabolism. However, we have found that several other worm HNF4-like receptors, including those previously implicated for affecting overall fat storage [12], do not significantly impact the expression of fatty acid metabolism genes (M. R. Van Gilst and K. R. Yamamoto, unpublished data). Thus, the regulation of fat metabolism does not appear to be a general mechanism common to all of the divergent NHRs; whether or not *nhr-49* is unique in its control of C. elegans fat metabolism remains to be determined.

The mechanism by which *nhr-49* influences fat storage is likely to be through modulation of β-oxidation gene expression (Figure 6). Overexpression of *acs-2* alone was sufficient to suppress the high-fat phenotype of *nhr-49(nr2041),* indicating that *acs-2* expression is rate-limiting in *nhr-49* deletion mutants, thus leading to reduced fat breakdown and excessive fat accumulation. Acyl-CoA synthetases activate fatty acids for many different metabolic pathways; in particular, mitochondrial acyl-CoA synthetases are likely to activate fatty acids for transport into the mitochondrial matrix, where the fatty acids are then subject to β-oxidation [18,19]. Notably, mitochondrial acyl-CoA synthetases are commonly regulatory targets for factors that control fat consumption [18]. We propose that by stimulating *acs-2* expression, *nhr-49* likely acts by increasing the flow of fatty acids into the mitochondria for β-oxidation (Figure 6).

Out of 11 *acs* genes in *C. elegans,* five of which are predicted to be mitochondrial, *nhr-49* specifically affected the expression of only *acs-2,* suggesting that *nhr-49* may be promoting transport of a specific subset of fatty acids into mitochondria. These subsets may differ by fatty acid chain length or tissue distribution. As both *acs-2* and *nhr-49* are expressed in numerous C. elegans tissues, regulation of fat consumption by *nhr-49* may occur throughout the animal.

Although *acs-2* is related (with approximately 25% identity) to multiple mammalian acyl-CoA synthetases, mammals contain a particular homolog with approximately 41% identity to the C. elegans ACS-2 protein; this mammalian ACS protein is yet to be characterized. It will be interesting to determine whether the mammalian counterpart of *acs-2* also serves as an important focal point for gene networks that regulate fat utilization in mammals.

Interestingly, we also found that *nhr-49* stimulates expression of a carnitine palmitoyl transferase (F09F3.9). Carnitine palmitoyl transferases execute the step immediately downstream of acyl-CoA synthetase in mitochondrial β-oxidation, directly shuttling activated acyl-CoAs into the mitochondrial matrix. Thus, *nhr-49* appears to target multiple enzymes involved in transporting fatty acids across the mitochondrial membrane for β-oxidation (Figure 6). RNAi of F09F3.9 did not detectably alter overall fat content, however, suggesting that interference of F09F3.9 expression alone is not sufficient to affect overall fat consumption under the conditions surveyed in our study.

In contrast, we found that RNAi of *ech-1,* another β-oxidation gene targeted by *nhr-49,* was sufficient to increase overall fat content; thus it is likely that the reduced expression of *ech-1* also contributes, in some fashion, to the high-fat phenotype of *nhr-49(nr2041).* Thus, we have demonstrated that *nhr-49* strongly promotes the expression of three separate enzymes predicted to participate in mitochondrial β-oxidation. Consequently, we suggest that *nhr-49* plays a prominent role in promoting the breakdown of fatty acids in mitochondria, and that, through control of β-oxidation, *nhr-49* influences overall fat storage.

In addition to its effect on β-oxidation, we found that *nhr-49* helps to maintain the balance of saturated and monounsaturated fatty acids. By stimulating the expression of three fatty acid Δ9-desaturase genes, *nhr-49* facilitates the conversion of saturated fatty acids to monounsaturated fatty acids, and consequently, *nhr-49(nr2041)* mutant animals are high in saturated fat and lower in monounsaturated fat. In *C. elegans,* the FAT-6 and FAT-7 SCDs may also be involved in the synthesis of PUFAs [17]. However, a dramatic reduction in *fat-7* expression, either by *nhr-49(nr2041)* deletion or by *fat-7* RNAi, did not significantly affect PUFA synthesis. This result suggests that a normal level of FAT-7 expression is not necessary for PUFA synthesis, likely because FAT-6 is the principle enzyme in this pathway, or because FAT-6 is capable of substituting for reduced FAT-7 activity. In either event, we propose that the primary effect of *nhr-49* on fatty acid composition is a modulation of the ratio of C18:0 to C18:1n9 fatty acid (Figure 6). However, we cannot rule out the possibility that *nhr-49* and *fat-7* also affect PUFA synthesis in a more localized manner, for example, specific cell types, such that changes in PUFA abundance are not detected in our whole-worm analysis.

Our results also indicate that the short life-span phenotype of *nhr-49(nr2041)* is due, at least in part, to reduced expression of *fat-7.* The strong correlation between the increase in C18:0 saturated fatty acid and the shortening of life span is compelling. Several reports in mammals have described changes in fatty acid composition during aging, and it has been argued that an alteration of fatty acid saturation ratios in mitochondrial membranes may affect the rate of aging [20]. Additionally, imbalance of fatty acid saturation has also been linked to numerous pathological conditions [15,16]. Although SCDs have not yet been linked to life span in mammals, studies in C. elegans have identified *fat-7* as one of the targets of the mechanisms that extend longevity through the insulin pathway [21]. Thus, it will be interesting to determine if the shortened life-span phenotype of *nhr-49(nr2041)* and *fat-7 RNAi* animals results from an improper ratio of saturated and monounsaturated fats, and if the mechanistic cause of the reduced life span is related to accelerated aging or to another pathology.

Although it is clear that *nhr-49* independently promotes the expression of genes in fatty acid β-oxidation and desaturation, we found that downstream components of these pathways do indeed communicate with each other. By inhibiting the expression of genes involved in β-oxidation, including the *nhr-49*-dependent genes *acs-2* and *ech-1, fat-7* works against the actions of *nhr-49,* acting to hinder fat consumption (Figure 6). Thus, our results reveal an elegant feedback mechanism: *nhr-49* induces the expression of β-oxidation enzymes—thus promoting fat consumption—yet by independently stimulating *fat-7* expression, *nhr-49* tempers the expression of the very same β-oxidation genes (Figure 6). Thus, we suggest that *nhr-49* serves as a key regulator of fat usage, governing pathways that consume fat for energy and partition fat for storage, potentially shifting the balance in response to changing energy needs. Indeed, we have found that in response to starvation, *nhr-49* selectively modulates the expression of these two pathways in order to increase fat consumption (M. R. Van Gilst and K. R. Yamamoto, unpublished data).

As the predominant function of *fat-7* is to convert C18:0 to C18:1n9 fatty acid, it is tempting to speculate that one of these two fatty acid species serves as a signal to modulate the feedback effect of *fat-7* on fatty acid β-oxidation (Figure 6). In this scenario, C18:0 could be serving as an activator of β-oxidation gene expression, C18:1n9 could be functioning as a repressor, or both (Figure 6). Although it seems reasonable to propose that an NHR would communicate this fatty acid signal, we found that the effects of *fat-7* on fatty acid β-oxidation were not dependent on *nhr-49;* hence, one of the other 283 C. elegans NHRs may mediate this effect.

Despite its strong sequence similarity with the mammalian HNF4 receptors, the overall biological effects of *nhr-49* on metabolism, fat storage, and life span are remarkably similar to those of the mammalian PPARs. Perhaps most striking is the finding that *nhr-49* and the PPARs, particularly PPARα and PPARδ, positively influence similar genes in multiple metabolic processes, including fatty acid β-oxidation, fatty acid desaturation, and fatty acid binding/transport [22,23]. Moreover, knockout of PPARα or PPARδ can lead to high-fat phenotypes [23,24], demonstrating that the physiological effects of these receptors on fat storage are similar to those of *nhr-49.*

The regulation of SCDs by *nhr-49* also parallels the regulation of the SCD1 gene by PPARα. In mammals, the Δ9-desaturase SCD1 is a lipogenic enzyme that is controlled by several key regulators of fat storage, including PPARα, leptin, and sterol response element-binding protein [15,25,26]. When SCD1 expression is left unchecked, as occurs in leptin knockout mice, severe obesity can result. Similar to what we found for the *C. elegans fat-7* gene, mammalian SCD1 affects fat storage by inhibiting β-oxidation [27]. Thus, because PPARα also promotes fat consumption by stimulating β-oxidation gene expression[28], it is clear that PPARα is independently regulating pathways that promote fat consumption and stimulate fat storage. In fact, a similar feedback mechanism to what we have described here for *nhr-49* has been proposed for PPARα and SCD1 [27,29]. Taken together, these results demonstrate that the multicircuited regulatory networks governing fat usage appear well conserved between *C. elegans nhr-49* and the mammalian PPARα.

Although we have not yet determined whether or not the *nhr-49*-dependent genes identified in this study are direct transcriptional targets, each is homologous to known transcriptional targets of the PPARs [22,23]. Because NHR-49 and the PPARs are clearly regulating similar physiological processes and gene networks, we believe that they are likely to be using similar molecular mechanisms, including direct transcriptional regulation. However, it is possible that *nhr-49* controls fatty acid β-oxidation and desaturation through an indirect mechanism, functioning upstream of the transcription factors that directly act on the *nhr-49*-dependent genes identified in this study. The latter possibility would also be intriguing, as it would demonstrate NHR involvement in fat regulation at a point upstream of a PPAR-like factor.

As the physiological functions and regulatory targets of *nhr-49* are highly similar to those of the mammalian PPARs, it seems reasonable to speculate that, like the PPARs, NHR-49 may be regulated by fatty acid ligands. By binding directly to ingested lipids and/or their metabolic products, NHR-49 could serve both as a sensor of intracellular fatty acid levels and an effector, responding to changes in fatty acid composition through modulation of fatty acid β-oxidation and desaturation. Interestingly, the mammalian HNF4 receptors, whose LBDs share 33% identity with the NHR-49 LBD, can bind to several saturated and monounsaturated fatty acids, including stearic and oleic acid [30,31]; the mammalian PPARs also bind to many types of fatty acids, although they interact preferentially with PUFAs [22]. It will be interesting to determine if mammalian HNF4 receptors harbor an undiscovered capacity to regulate fat metabolism in mammals, or if PPARs have adopted their regulatory functions by evolving from an ancestral HNF4 gene that had properties similar to *nhr-49* and the modern PPARs. Alternatively, the PPARs and *nhr-49* may have acquired their activities through convergent evolution.

Precise regulation of fatty acid β-oxidation and desaturation is crucial for physiological homeostasis; imbalance in these metabolic pathways in humans can lead to disorders such as diabetes, obesity, atherosclerosis, and accelerated aging. However, although NHRs are important targets for drugs that moderate these metabolic disorders, the NHR-mediated regulatory networks that govern fat usage are notably complex and poorly understood. We showed here that many features of *C. elegans nhr-49* regulation of fat metabolism are closely analogous to those of the mammalian PPARs, key targets for the treatment of metabolic disease. Moreover, our C. elegans studies revealed that *nhr-49,* like PPARα, oversees a branch point for two separate circuits within a network that controls fatty acid consumption and composition.

The extent of the functional homology between *nhr-49* and PPARα remains to be determined, that is, whether or not *nhr-49* also functions by binding to fatty acid ligands and directly regulating gene transcription. Nevertheless, our finding that *nhr-49* is a key regulator of fat metabolism in C. elegans represents a considerable advance in our understanding of NHR control of fat storage and composition in invertebrates. Consequently, we suggest that these and future studies in C. elegans will continue to enhance our understanding of the complex roles of NHRs and their ligands on gene networks governing fat physiology.

## Materials and Methods

### 

#### RNAi constructs

All of the RNAi constructs used in the NHR screen were created by cloning full-length NHR cDNAs into the vector L4440 (Andy Fire, Stanford University). The *fat-7* RNAi vector was a gift from Jennifer Watts (Washington State University). All of the other RNAi constructs were obtained from the Ahringer RNAi library [32].

#### Life span assays

Approximately 30–40 starved L1 worms were transferred to RNAi bacteria (HT115 transformed with RNAi clone) and life-span assays were carried out at 23 °C as described previously [33]. *nhr-49(nr2041)* and WT life-span assays were carried out at 23 °C on OP50 bacteria.

#### Nile Red assays

Nile Red fat-staining assays were carried out as described previously [12].

#### Selection of fatty acid and glucose metabolism genes

Most of the fatty acid and glucose metabolism candidate genes examined in this study were initially identified using the KEGG pathway database (http://www.genome.ad.jp/kegg/pathway.html). Other genes were identified using BLAST searches designed to find C. elegans open reading frames that were highly related to known mammalian glucose and fat metabolism enzymes, or to plant glyoxylate cycle genes.

For prediction of β-oxidation enzyme subcellular localization, we used the following online prediction tools. TargetP server version 1.01 (http://www.cbs.dtu.dk/services/TargetP/) [34] and PSORT (http://psort.nibb.ac.jp/form.html).

#### Preparation of nematode total RNA

Both C. elegans N2-Bristol (WT) and *nhr-49(nr2041)* were grown at 23 °C on high-growth plates seeded with OP50 bacteria. Gravid adults from 30 10-cm plates were bleached, and embryos were dispersed onto 15-cm nematode growth media (NGM)-lite plates seeded with OP50. The worms were distributed as follows: for L1 harvest, 80,000 embryos/plate; for L2 harvest, 40,000 embryos/plate; for L3 harvest, 20,000 embryos/plate; and for L4 harvest, 10,000 embryos/plate. The remaining embryos were saved for embryonic RNA preparation. Worms were harvested at the appropriate stage, washed twice with M9, and frozen in liquid nitrogen. For RNAi experiments, 10,000 embryos were placed onto NGM-lite plates containing 8 mM IPTG and 100 μg/ml carbenicillin seeded with control bacteria (HT115 transformed with empty L4440 vector) or *nhr-49* RNAi bacteria (HT115 transformed with L4440-*nhr-49*). Worms were harvested at the L4 stage of development, washed twice with M9, and frozen in liquid nitrogen. For RNA preparation, worms were thawed at 65 °C for 10 min, and RNA was isolated using the Tri-Reagent Kit (Molecular Research Center, Cincinnati, Ohio, United States). Isolated total RNA was subjected to DNAase treatment and further purification using RNAeasy (Qiagen, Valencia, California, United States).

#### QRT-PCR

cDNA was prepared from 5 μg of total RNA in a 100-μl reaction using the Protoscript cDNA preparation kit (New England Biolabs, Beverly, Massachusetts, United States). Primer pairs (primer sequences available upon request) were diluted into 96-well cell culture plates at a concentration of 3 μM. Next, 30-μl PCR reactions were prepared in 96-well plates. Each PCR reaction was carried out with *Taq*DNA Polymerase (Invitrogen, Carlsbad, California, United States) and consisted of the following reaction mixture: 0.3 μM primers, 1/500th of the cDNA reaction (corresponds to cDNA derived from 10 ng of total RNA), 125 μM dNTPs, 1.5 mM MgCl_2_, and 1X reaction buffer (20 mM Tris pH 8.4, 50 mM KCl). 0.15 μl (0.75 units) of *Taq*DNA Polymerase was used for each reaction. Formation of double-stranded DNA product was monitored using SYBR-Green (Molecular Probes, Eugene, Oregon, United States). All QRT-PCR reactions were carried out and analyzed on a DNA Engine-Opticon 2 (MJ Research, Waltham, Massachusetts, United States). Data were collected using RNA from at least three independent C. elegans growths. To determine the relationship between mRNA abundance and PCR cycle number, all primer sets were calibrated using serial dilutions of cDNA preparations. Primer sets were also calibrated by performing QRT-PCR reactions on serial dilutions of C. elegans genomic DNA. Relative abundance is reported as the mRNA abundance of each gene relative to the mRNA abundance of several control genes, which are expressed at constant levels throughout development.

#### GC/MS

Fatty acids were isolated from 10,000 L4 animals grown on a single 15-cm NGM-Lite plate. For WT and *nhr-49(nr2041)* experiments, worms were grown at 23 °C on a lawn of OP50. For RNAi, 10,000 embryos were placed on control bacteria, or on *fat-5, fat-7, ech-1, acs-2,* or *nhr-49* RNAi bacteria. Fatty acid extract was prepared as described previously [17]. GC/MS spectra were collected on an HP 6890 gas chromatograph outfitted with a J&W DB-XLB column. The mass spectrometer was an HP MSD 5973 (Agilent, Palo Alto, California, United States) and data were analyzed using Chemstation version A.03.00 software (Agilent). Peaks were assigned using fatty acid standards.

#### GFP reporter analysis and ectopic expression of ACS-2::GFP

To make the P*_nhr-49_*GFP plasmid, a genomic fragment including 3.5 kb of the *nhr-49* upstream sequence and 88 bp of the first exon of *nhr-49* was ligated into the L3691 GFP expression vector (a gift of A. Fire). Germ-line transformation was performed following standard procedures [35]. P*_nhr-49_*GFP was injected at the following concentrations: 1 ng/μl, 5 ng/μl and 20 ng/μl along with the co-injection marker pRF4 *rol-6(su1006)* injected at 60 ng/μl. At all three P*_nhr-49_*GFP concentrations, we were unable to obtain stably transmitting lines. Animals used for GFP imaging were derived from the F1 generation and were mosaic, hosting the P*_nhr-49_*GFP transgene in only a subset of tissues.

To construct *acs-2::gfp,* a 4-kb genomic fragment, including the full-length *acs-2* gene plus 2 kb of upstream sequence, was cloned into the vector L3691. Germ-line transformation was performed using 50 ng/μl of *acs:2::gfp* and 50 ng/μl pRF4 *rol-6(su1006).* For N2/*acs-2::gfp* and *nhr-49(nr2041)/acs-2::gfp,* over ten independent transmitting lines were obtained and analyzed by GFP microscopy. GFP reporter expression was observed using a Zeiss Axiovert S100 fluorescence microscope (Carl Zeiss, Thornwood, New York, United States) under a 40X objective.

For rescue experiments, 50–100 ng/μl of *acs-2::gfp* was injected into *nhr-49(nr2041)* and WT animals along with 50 ng/μl of pRF4 rol-6(su1006). Transgenic adults, selected for rolling behavior, were placed on Nile Red plates and assayed for fat content as described above. pRF4 *rol-6(su1006)* was injected into *nhr-49(nr2041)* in combination with unrelated plasmids to demonstrate that the lowered fat content was not a result of hosting a transgenic array or caused by expression of the *rol-6(su1006)* marker gene.

## Supporting Information

Table S1 Raw Data for QRT-PCR Screen Data are shown as C_t_, the number of cycles required for amplification of a particular target gene from a cDNA preparation. Changes in gene expression in the *nhr-49(nr2041)* mutant were measured as ΔC_t_. ΔC_t_ are obtained by subtracting *nhr-49*(C_t_) from N2(C_t_) and adjusting for RNA concentration differences with a correction factor (CF). The CF was determined by aligning the data to a set of standard genes. Thus the equation for ΔC_t_ is as follows:

Genes were designated as *nhr-49* dependent if the C_t_ was >1.0 in multiple developmental stages (and was greater than the standard error). *nhr-49* targets are marked with an X. The QRT-PCR results of the major *nhr-49* targets, *acs-2, ech-1, fat-5,* and *fat-7,* were confirmed with a second primer pair.
(100 KB XLS).

## Abbreviations

GC/MS: gas chromatography/mass spectrometry
GFP: green fluorescent protein
HNF4: hepatocyte nuclear factor 4
LBD: ligand binding domain
NGM: nematode growth media
NHR: nuclear hormone receptor
PCR: polymerase chain reaction. PPAR
PUFA: polyunsaturated fatty acid
QRT–PCR: quantitative RT-PCR
RNAi: RNA interference
SCD: stearoyl-CoA desaturase
WT: wild-type
